# Scaling Approach to Doniach Phase Diagram: Application to CeRu_2_Ge_2_ and EuCu_2_(Ge_1−*x*_Si_*x*_)_2_

**DOI:** 10.3390/ma18163755

**Published:** 2025-08-11

**Authors:** Veljko Zlatić, Ivica Aviani

**Affiliations:** 1 Institute of Physics, Bijenička Cesta 46, P.O. Box 304, 10000 Zagreb, Croatia; 2 Faculty of Science, University of Split, Ruđera Boškovića 33, 21000 Split, Croatia

**Keywords:** Doniach phase diagram, heavy fermions, Anderson model, scaling solution

## Abstract

We calculate the Doniach phase diagram of heavy-fermion systems containing Ce and Eu ions, using the scaling solution of the periodic Anderson model, and compare the results with the experimental data on CeRu_2_Ge_2_ and EuCu_2_(Ge_1−*x*_Si_*x*_)_2_. The temperature–pressure (T–p) phase diagram emerges from the competition between the pressure-dependent Kondo interaction and the temperature- and pressure-dependent RKKY interaction. Both are derived using scaling equations in the presence of crystal-field effects: Kondo temperature $T_{K}$ is related to the coupling constant g(p), where p is the control parameter, and the temperature-dependent renormalized coupling $g(T,T_{K}\left(g\right))$. For comparison with the experiment, we assume a linear dependence of g on the control parameter, which could be pressure or composition. The Néel temperature $T_{N}\left(p\right)$ is obtained by comparing the free energies of the system in the antiferromagnetic and paramagnetic states. The resulting asymmetric $T_{N}\left(p\right)$ arises naturally from the exponential growth of $T_{K}\left(p\right)$ and a much slower polynomial growth of the RKKY interaction. Phase diagrams for CeRu_2_Ge_2_ and EuCu_2_(Ge_1−*x*_Si_*x*_)_2_ successfully capture key experimental features: pressure-induced suppression of magnetic order, the peak of RKKY interaction energy, and crossover to a heavy-Fermi-liquid regime at high coupling strength. Our work provides the first quantitative, material-specific construction of Doniach diagrams, clarifies the entropy removal at low temperatures and offers predictive insight for future experiments under extreme conditions.

## 1. Introduction

Heavy fermion materials are a class of intermetallic compounds that contain rare earths or actinides and typically occur in Ce-, Yb-, Eu- and U-based compounds with partially filled 4f or 5f shells [1,2]. Despite the diversity of their chemical composition and structure, they all share a basic microscopic feature: the entanglement of localized f electrons with itinerant conduction electrons which drives the Kondo effect and Ruderman–Kittel–Kasuya–Yosida (RKKY) interaction [3,4]. This leads to unusual properties at low temperatures, such as an enhanced effective electron mass, non-Fermi liquid behavior, and unconventional superconductivity [5,6]. Many heavy fermion materials exhibit quantum phase transitions at zero temperature, and their tunability by external pressure, magnetic field, or chemical substitution makes them exceptional systems for fundamental quantum materials research, including studies of strong correlations, quantum phase transitions, and emergent electronic ordering [7,8,9]. At high temperatures, the f electrons in these systems act as localized magnetic moments which scatter conduction electrons, giving rise to the Kondo effect. As the temperature is lowered, this scattering leads to the screening of local moments and, in the case of a single magnetic impurity, it eventually gives rise to the formation of a singlet ground state [4]. In a lattice of such screened moments, the coherence emerges below a characteristic temperature T^*^, resulting in the formation of a heavy Fermi liquid with a large Fermi surface that includes f electrons [2,8]. The term “heavy fermion” arises because the effective mass of quasiparticles in these materials can be 100–1000 times that of a free electron [1]. Conduction electrons also mediate an indirect exchange interaction between local moments, known as the RKKY interaction, which favours a long-range magnetic ordering [3].

The competition between Kondo screening (favouring a non-magnetic ground states) and RKKY interaction (favouring magnetism) determines the ground state of the system and Doniach phase diagram captures the main feature of this interplay: an increase in the Kondo interaction J_*K*_ drives a transition from an antiferromagnetically ordered phase with a small Fermi surface, to a paramagnetic heavy Fermi liquid with a large Fermi surface. The transition is marked by a quantum critical point (QCP) [3,7,10].

Since its introduction [3], the Doniach diagram has widely been envoked to interpret qualitative features of magnetic and non-magnetic ground states of Ce- and Eu-based intermetallics. Over the past few decades, extensive experimental investigations have revealed complex phase diagrams under pressure, temperature, and magnetic field [11,12,13,14]. These works have outlined general systematics and surprising deviations in (p,T,H) phase space, including non-Fermi-liquid behavior near quantum critical points.

Early experimental studies, such as those by Holland-Moritz et al. [15], provided neutron scattering and spectroscopic insights into the hybridization and valence fluctuations underpinning these phenomena. Bauminger et al. were the first to discover charge fluctuations in EuCu_2_Si_2_ using Mössbauer spectroscopy [16]. Hossain et al. investigated a series of EuCu_2_(Ge_1−*x*_Si_*x*_)_2_ solid-state alloys and found a transition from an antiferromagnetic phase to a fluctuating valence phase and a heavy fermionic behaviour with increasing Si content [17]. The parallel development of Ce-based systems has provided an important context for understanding pressure- and composition-induced transitions in rare-earth intermetallics. Süllow et al. and Wilhelm et al. investigated the Doniach phase diagram in pressurised CeRu_2_Ge_2_ and showed a continuous evolution from the magnetic order to the Fermi liquid regime [11,12] and the occurrence of intermediate valence behaviour [12].

More recently, Iha et al. [18] reported a detailed experimental study of the T–x phase diagram of CeCu_2_(Ge_1−*x*_Si_*x*_)_2_ and noted its consistency with Doniach-type behavior, though without a quantitative theoretical model. While these studies established a rich phenomenology and qualitative trends in heavy fermion phase diagrams, the Doniach diagram was typically invoked in a schematic way.

Theoretically, the competition between the Kondo interaction and the RKKY interaction, and its effect on the phase diagram of HFs, has initially been studied using the periodic Kondo model [3,19,20,21,22] with the ensuing Doniach diagram providing a foundational framework for understanding that interplay [1]. Subsequent theoretical studies of quantum criticality have used the Anderson model to expand the Doniach concept, so as to include valence fluctuations, disorder, and underscreened Kondo effects [2,23]. Several works have attempted semi-quantitative treatments by plotting experimental phase boundaries alongside model curves. For example, Matsumoto et al. [22] analyzed Ce-based 122 compounds using DFT+DMFT to place real systems on a generalised Doniach diagram, but did not numerically reproduce full T–p curves. More recently [24], the competition between various interactions and the ensuing quantum criticality in the periodic Anderson model has been studied using cellular dynamical mean-field theory, with the numerical renormalization group as a cluster impurity solver.

By introducing two essential energy scales, the Kondo temperature T_*K*_ and the RKKY interaction strength $T_{RKKY}$, the Doniach diagram provides a foundational understanding of the interaction between localized moments and conduction electrons. While simplified, it serves as an important entry point into more complex behaviour. To account for additional factors such as frustration, valence fluctuations, or non-Fermi liquid behaviour, the so-called global phase diagram extends Doniach’s diagram by adding a second tuning parameter (e.g., quantum fluctuations or dimensionality), thereby allowing for a deeper understanding of exotic phases and multiple quantum critical points that occur in these systems [8,9,25,26].

Heavy fermion compounds such as CeRu_2_Ge_2_ or CeCu_2_Ge_2_ under pressure [27] and CeCu_2_(Ge_1−*x*_Si_*x*_)_2_ or EuCu_2_(Ge_1−*x*_Si_*x*_)_2_ or EuPd_2_(Ge_1−*x*_Si_*x*_)_2_ with varying Si concentrations [18,28,29,30] are ideal platforms to study the competition between Kondo screening and magnetic ordering. In both cases, tuning an external parameter (pressure in CeRu_2_Ge_2_ and CeCu_2_Ge_2_) or chemical substitution in CeCu_2_(Ge_1−*x*_Si_*x*_)_2_, EuCu_2_(Ge_1−*x*_Si_*x*_)_2_ and EuPd_2_(Ge_1−*x*_Si_*x*_)_2_, modifies the strength of the Kondo coupling J_*K*_ and drives the system to a magnetic or paramagnetic ground state. These observations align well with the Doniach framework, which relates the principal energy scales T_*K*_ and T_*RKKY*_ to the observed phase transitions.

**Figure 1:**
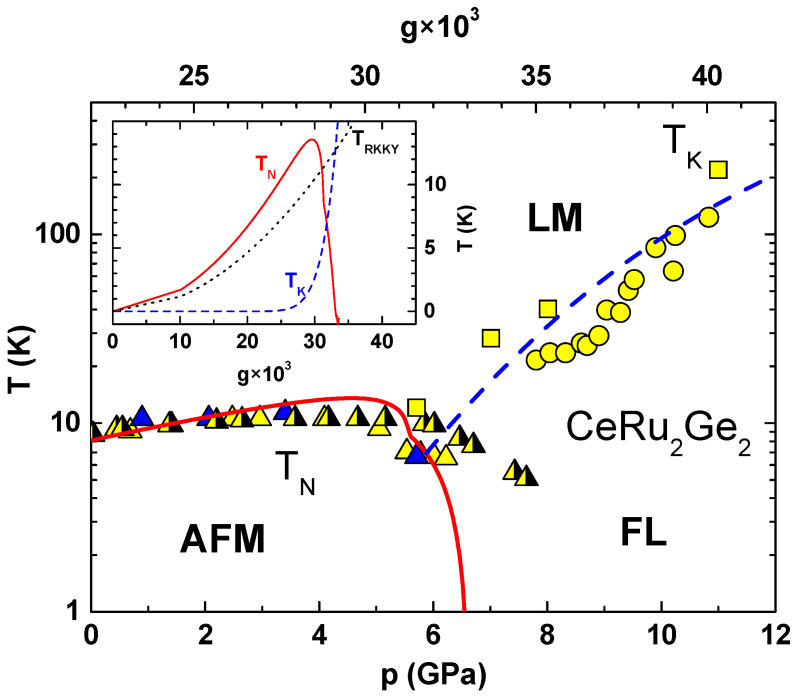
The Kondo scale $T_{K}\left(p\right)$ of CeRu_2_Ge_2_ obtained from data on ρ(T) (squares) and α(T) (circles), and the Neel temperature $T_{N}\left(P\right)$ obtained from the data on ρ(T) (half-filled triangles), calorimetric data (yellow triangles), and combined ρ(T) and α(T) data (blue triangles) are plotted versus pressure [34]. The full and long-dashed lines show $T_{N}\left(p\right)$ and $T_{K}\left(p\right)$ defined by Equations (1) and (5), respectively. The inset shows $T_{K}\left(p\right)$, $T_{N}\left(p\right)$, and $T_{RKKY}\left(p\right)$ defined by Equation (2) (dashed line) and plotted versus g(p). The model parameters used for the plot and the p→g mapping which defines the upper abscissa are explained in the text.

**Figure 2:**
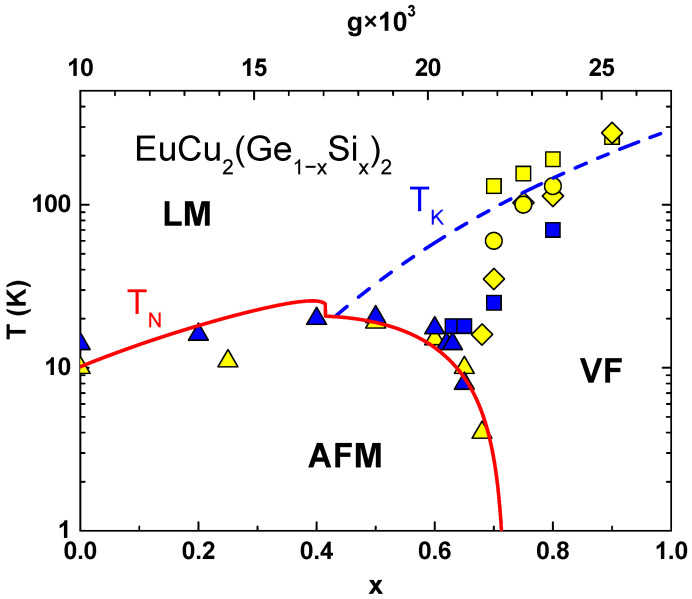
The experimental magnetic phase diagram of EuCu_2_(Ge_1−*x*_Si_*x*_)_2_. is compared with calculated Kondo temperature $T_{K}$ (dashed line) and the magnetic ordering temperature $T_{N}$ (full line). The yellow symbols are the data from ref. [33] and the blue symbols the data from ref. [17]. The Néel temperature (triangles) is obtained from the specific heat anomaly. The Kondo temperature is estimated from the thermopower (squares), resistivity (diamonds), and from the X-ray absorption spectra (circles). The interaction strength $g=\Gamma /\pi E_{f}$ used to calculate $T_{K}$ and $T_{N}$ from Equations (1) and (5), respectively, is shown on the upper abscissa.

The functional form of the response functions of HFs depends on the relative importance of these couplings which are easily changed by a control parameter, *p*, i.e., by pressure, chemical pressure, or magnetic field [3,4,6,17,31,32,33,34] The data analysis yields the Kondo temperature $T_{K}\left(p\right)$ and Néel temperature $T_{N}\left(p\right)$ and plotting them against the control parameter yields the Doniach diagram. It separates the phase space into several characteristic regions [3,4], as shown by Figure 1 in the case of CeRu_2_Ge_2_ [34], with pressure as the control parameter, and in Figure 2 for EuCu_2_(Ge_1−*x*_Si_*x*_)_2_ [33], with chemical pressure as the control parameter. Similar behaviour is also found in CeCu_2_(Ge_1−*x*_Si_*x*_)_2_, EuPd_2_(Ge_1−*x*_Si_*x*_)_2_, and several other HF materials with RE ions [17].

Various phase-space regions appearing in the Doniach diagram exhibit the following characteristic features. In the high-temperature phase, the RE ions behave as independent LM and all the properties of the system are determined by its pressure-dependent Kondo temperature $T_{K}\left(p\right)$. The resistivity is a logarithmic function of $T/T_{K}$, the susceptibility is Curie–Weiss like with $\theta ≃T_{K}$, the magnetic moment of 4*f* electrons is close to what one finds in a free Ce ion, and the entropy is dominated by a large paramagnetic contribution, $S_{f}≃k_{B}\ln N$, where *N* is the effective degeneracy of the LM in a given temperature range [35]. Experimentally, $T_{K}\left(p\right)$ is either obtained from transport measurements, like thermopower α(T) or electrical resistivity ρ(T), or it is defined by temperature at which the entropy drops to half of its high-temperature value [17,33,34]. The overall dependence of $T_{K}\left(p\right)$ on the control parameter is rather smooth, even though the experimental values of $T_{K}\left(p\right)$ inferred from different measurements are not exactly the same. Theoretically, $T_{K}$ is defined as the scaling invariant of the Anderson model which we use to analyze the data.

At low temperatures, a large paramagnetic entropy of LM cannot be sustained but the mechanism by which the entropy is removed from the system and the nature of the ensuing ground state (GS) depend on the relative magnitude of the Kondo and RKKY interactions. We distinguish two limiting cases: $T_{K}\gg T_{RKKY}$ and $T_{K}\ll T_{RKKY}$, where $k_{B}T_{RKKY}=E_{RKKY}$ is the energy gain due to the antiparallel alignment of the neighbouring LMs caused by the RKKY interaction (in what follows, we set $k_{B}=1$). The RKKY temperature is related to $T_{K}$ and $T_{N}$ but, unlike these temperatures, it is not directly discernible in the experimental data. For a given heavy fermion compound, the value of $T_{RKKY}\left(p\right)$ is estimated a posteriori by model calculations (see Equation (2)). If at ambient or low pressure we have $T_{K}\left(p\right)<T_{RKKY}\left(p\right)$, the low-entropy state is reached by an AFM transition at temperature $T_{N}\left(p\right)$. On the other hand, if we have $T_{K}\left(p\right)>T_{RKKY}\left(p\right)$, the paramagnetic entropy is not eliminated by an AMF transition but rather by a crossover from a LM phase to a heavy fermi liquid (FL). The temperature of the crossover is proportional to $T_{K}\left(p\right)$ and the high-pressure behaviour is similar to what one finds for an isolated Kondo impurity: at low temperatures, $T\ll T_{K}$, the conduction electrons screen the LM by forming a Kondo singlet and the ensuing GS is a non-degenerate FL [4]. The two ground states are separated by a QCP.

The presented approach aims to construct a quantitative realization of the Doniach scenario by solving the scaling equations of the Anderson model and mapping the results directly onto experimental pressure- and doping-dependent transition temperatures. It provides a coherent and computationally accessible framework for the calculation of the T–p and T–x phase diagrams of heavy fermion systems. As illustrated in the case of CeRu_2_Ge_2_ and EuCu_2_(Ge_1−*x*_Si_*x*_)_2_, by linking a simple theoretical model (Doniach picture) with experimentally measurable parameters, we provide a practical tool for the prediction and description of magnetic transitions and quantum critical points in f-electron systems. This has implications for the understanding of quantum criticality, magnetic ordering, and unconventional superconductivity in strongly correlated electron materials.

The behaviour of Yb-based intermetallics, in which the coupling constant is a decreasing function of the control parameter, was explained using the same approach in Ref. [32]. Here, we discuss the cerium- and Europium-based intermetallics [17,33,34] in which the coupling constant is an increasing function of the control parameter. The model takes into account the charge transfer between the 4*f* and *c*-states, which is important at high pressure, and also considers the crystal-field (CF) splitting, which makes the effective degeneracy of the 4*f*-states pressure- and temperature-dependent.

Unlike most previous studies, our paper provides a quantitative realization of the Doniach picture: we numerically solve the scaling equation for the Kondo temperature T_*K*_(g), where g(p) is the coupling constant of the Anderson model [4], compute the Néel temperature T_*N*_(g) from a temperature-dependent RKKY interaction, and map these results onto experimental pressure or doping axes using simple linear coupling functions g(p) or g(x). To the best of our knowledge, this is the first application of the Doniach framework that quantitatively reproduces experimental phase boundaries over the entire T–p and T–x range for CeRu_2_Ge_2_ and EuCu_2_(Ge_1−*x*_Si_*x*_)_2_.

The paper is organised as follows. First, we introduce the model and the scaling solution and provide the relationship between the coupling constant g(p) and the scaling invariant $T_{K}\left(p\right)$. This reveals the central feature of the Kondo effect, namely, the exponential dependence of $T_{K}\left(p\right)$ on g(p). The scaling law also yields the renormalised, temperature-dependent coupling constant, g(p,T), which is used in the renormalised perturbation theory to study the properties of the LM phase. By matching the theoretical and experimental values of $T_{K}\left(p\right)$, we obtain the dependence of g(p) on the control parameter and calculate $T_{RKKY}\left(p\right)$ of a given compound. Once we have $T_{K}\left(p\right)$, g(p), and g(p,T), we can compute the free energy of the LM phase. With $T_{RKKY}\left(p\right)$ at hand, we estimate the free energy of the AFM phase, and by comparing it with the free energy of the LM phase we find the pressure dependence of $T_{N}\left(p\right)$. Finally, the theoretical results are used to discuss the phase diagram of CeRu_2_Ge_2_, EuCu_2_(Ge_1−*x*_Si_*x*_)_2_, and similar compounds with the RE ions.

## 2. Model and Calculation

The periodic Anderson model with the CF split 4*f* states is characterized by the unperturbed *c*-band of width *D*, the unrenormalized excitation energy of the 4*f* states $E_{f}$, the energy gain due to the hybridization of the 4*f* states with conduction electrons, Γ(p), and the degeneracies of the crystal-field split 4*f* states. (In the case of independent 4*f* ions, Γ(p) is simply the width of the virtual bound state.) We consider the model in which the number of *f*-electrons per site is $n_{f}$, the number of *c*-electrons is $n_{c}$, and assume an infinite *f*-*f* correlation, so that the 4*f* state can only be singly occupied or unoccupied. The degeneracies of the CF states are determined by the point-group symmetry of the crystal, while the neutron scattering or magnetization data provide the splittings. For a given $n_{c}$, $n_{f}$, and the CF splitting, the low-energy excitations of the model depend in an essential way [4] on the dimensionless coupling constant $g\left(p\right)=\Gamma \left(p\right)/\pi |E_{f}|$.

In Ce and Eu compounds, g(p) increases with pressure and all the properties change drastically due to the exponential dependence of $T_{K}$ on g(p), as discussed in Ref. [35]. The electrical resistance of heavy fermion compounds in the LM region of the phase space is large, so that we can treat the 4f ions as incoherent Kondo scatterers. In that case, the Kondo scale of the model, assuming $n_{f}≃1$, can be related to the coupling constant by the ‘poor man’s scaling’ [36,37]. For two excited CF levels which are $N_{1}$- and $N_{2}$-fold degenerate, and separated from the $N_{0}$-fold degenerate CF ground state by energies $\Delta_{1}$ and $\Delta_{2}$, we have the scaling equation [36,37,38,39],

(1) $$\begin{array}{c} g\left(p\right)\exp\left[-\frac{1}{g(p)}\right]={\left(\frac{T_{K}\left(p\right)}{D}\right)}^{N_{0}}{\left(\frac{T_{K}\left(p\right)+\Delta_{1}}{D+\Delta_{1}}\right)}^{N_{1}}{\left(\frac{T_{K}\left(p\right)+\Delta_{2}}{D+\Delta_{2}}\right)}^{N_{2}} \end{array}.$$

This equation holds in the LM regime and to describe CeRu_2_Ge_2_, which we take as a case study, we assume $N_{0}=N_{1}=N_{2}=2$, $\Delta_{1}=500$, and $\Delta_{2}=750$ K [40].

At a given pressure, the properties of the model are calculated by the lowest-order (renormalized) perturbation theory with an effective temperature-dependent coupling constant g(p,T), which is obtained from Equation (1) by rescaling the *c*-bandwidth down to D≃T [41]. This is equivalent to summing up the most diverging diagrams of the perturbation expansion in terms of the bare coupling and yields the correlation functions which are universal functions of $T/T_{K}$ [41]. The results obtained by the renormalized perturbation theory are in a qualitative agreement with the NCA [35,37] and the NRG calculations [4,42,43], which also show that the scaling law holds not only for $T\geq T_{K}\left(p\right)$ but can be extended down to $T<T_{K}\left(p\right)$ and it only ceased to be valid for $T\ll T_{K}\left(p\right)$.

The scaling equation allows us to estimate the pressure dependence of g(p) in the following way. We take the experimental values of $T_{K}\left(p\right)$ at two different pressures, $p_{1}$ and $p_{2}$, find the corresponding bare couplings by solving Equation (1), and define g(p) for $p_{1}<p<p_{2}$ by a linear interpolation. The upper abscissa of the main panel in Figure 1 shows g(p) obtained for CeRu_2_Ge_2_ in such a way, while the inset shows $T_{K}\left(p\right)$ plotted as a function of g(p) (long-dashed line). The near-exponential dependence of $T_{K}\left(p\right)$ on g(p) is typical of Kondo physics and explains the extreme sensitivity of heavy fermions on the control parameter (pressure, chemical pressure, or magnetic field).

In addition to the on-site Kondo coupling, the hybridization between the 4*f* and *c*-states also gives rise to the RKKY spin-density oscillations in the *c*-band. This spin density couples to the LMs at the neighbouring sites and, if strong enough, it prevents the spin–flip scattering and inhibits the Kondo effect. The energy gain due to the RKKY coupling is calculated by the 2nd-order perturbation theory in terms of the bare coupling [44]. For Ce ions surrounded by *z* neighbours at points **r**, this gives

(2) $$\begin{array}{c} E_{RKKY}\left(g,r\right)=18\pi zS\left(S+1\right)\left|F\left(2r\cdot k_{F}\right)\right|D\;\times \;g^{2}, \end{array}$$

where *S* is the angular momentum of the lowest CF state, $F\left(\eta \right)=\left[-\eta \cos\eta +\sin\eta \right]/\eta^{4}$ is the oscillating function, $k_{F}$ is the Fermi momentum of unperturbed *c*-electrons and g is the unrenormalized coupling constant. For a material with a given $T_{K}\left(p\right)$, the pressure-dependent coupling constant which enters in Equation (2) is obtained by solving numerically Equation (1). Finally, the boundary between various characteristic phases of a heavy fermion is found by equating their free energies.

The spin-density oscillations induced by the RKKY coupling follow, like Friedel charge density oscillations, from the Fermi-edge discontinuity of the electron distribution function. Thus, they are temperature-dependent and can be neglected at high temperatures [45]. At high pressure, where g(p) is large and $T_{K}\left(p\right)$ is huge, the paramagnetic entropy is eliminated by the Kondo effect and the RKKY interaction does not play any role. For $T_{RKKY}\ll T\ll T_{K}$, the screening of local moments gives rise to the LM-FL crossover. On the other hand, the values of $T_{K}\left(p\right)$ at low pressure decrease exponentially with g(p), so that the RKKY coupling, which is a parabolic function of g(p), dominates for $T_{K}<T<T_{RKKY}$. The magnetic field due to the RKKY oscillations inhibits the Kondo effect and, if strong enough, it quenches the Kondo scattering and stabilises the LM on the neighbouring sites before the Kondo singlets are formed. In that case, the large entropy of the paramagnetic state is removed at low temperatures by the formation of a magnetically ordered Néel state. Since Kondo scattering is absent in the magnetically ordered phase, $E_{RKKY}$ in Equation (2) is calculated with unrenormalised g(p).

The free energy of the Néel state is given by

(3) $$\begin{array}{c} F_{N}=E_{c}+E_{f}-E_{RKKY}, \end{array}$$

where $E_{c}$ and $E_{f}$ are the unperturbed internal energies of *c* and *f* electrons, respectively, and $E_{RKKY}$ approximates the energy gain due to the alignment of 4*f* moments on the neighbouring sites, as given by Equation (2). The above expression neglects the entropy of magnetic excitations which one can find in the AFM phase.

The free energy in the LM regime is

(4) $$\begin{array}{c} F_{LM}=E_{c}+E_{f}-E_{fc}-TS_{LM}, \end{array}$$

where $E_{fc}$ is the energy gain due to hybridization and $S_{LM}$ is the LM entropy. The renormalized perturbation theory gives $E_{fc}\left(T\right)=\langle H_{cf}\rangle ≃g\left(p,T\right)T_{K}$, where $H_{cf}$ is the interacting part of the Hamiltonian and g(p,T) is obtained from Equation (1) at D=T. For $T\gg T_{K}$, the effective coupling is small, g(p,T)≪1, and the entropy is close to the free-ion value, $S_{LM}≃S_{f}$. At lower temperatures, the effective coupling and $E_{fc}$ grow logarithmically, while the entropy decreases. The renormalized perturbation theory yields the approximate relation $S_{LM}≃\left(1-g^{3}\right)S_{f}$ [41].

If the paramagnetic entropy of the LM phase is removed by the magnetic transition, the Néel temperature $T_{N}$ follows from the condition $F_{N}=F_{LM}$, such that $E_{RKKY}=E_{fc}+T_{N}S_{LM}$. This gives

(5) $$\begin{array}{c} T_{N}\left(g\right)=\frac{E_{RKKY}\left(g\right)-g\left(p,T_{N}\right)E_{K}\left(g\right)}{S_{LM}}, \end{array}$$

where $S_{LM}$ is the paramagnetic entropy which we approximate by $S_{LM}≃S_{f}$. A unique determination of $T_{N}$ for a particular compound requires the value of $k_{F}$ in the argument of the oscillating function in Equation (2). Since this is not known, we adjust the amplitude of $F(2{r\cdot k}_{F})$, so that the Néel temperature at ambient pressure matches the experimental result. (Our choice satisfies $F\left(\eta \right)\geq F_{min}$, where $F_{min}=-5.06\;\times \;10^{-3}$ is the absolute minimum of the oscillating function.)

The theoretical calculation and the comparison with the experimental results can be summarised as follows. The T–p phase diagram arises from the competition between Kondo and RKKY interactions, which depend on pressure and temperature. In our theoretical model, these interactions are governed by the coupling constant *g*, which is assumed to be a linear function of pressure *p*. Consequently, the comparison between theoretical predictions and experimental data is carried out through this pressure dependence g(p). To compute the Néel temperature $T_{N}$ according to Equation (5), the following quantities are evaluated as functions of the coupling constant *g*: the RKKY energy $E_{RKKY}\left(g\right)$, the Kondo temperature $T_{K}\left(g\right)$, and the renormalized temperature-dependent coupling constant $g_{R}\left(T,g\right)$. The magnetic entropy change is approximated as $S_{LM}=k_{B}\ln\left(N_{0}\right)$, where $N_{0}$ is the ground-state degeneracy of the localized electrons.

The RKKY energy $E_{RKKY}\left(g\right)$ is obtained from Equation (2). For the lowest crystal electric field (CEF) doublet of the cerium ion (with spin S=1/2), and using the parameters D=4 eV, z=6, and $F\left(\eta \right)=-9.824\;\times \;10^{-4}$, we find: $E_{RKKY}\left(g\right)=1.000\cdot g^{2}$ eV. The value of F(η) is adjusted to ensure the theoretical values of $T_{N}$ best fit the experimental data. The Kondo temperature $T_{K}\left(g\right)$ is calculated by numerically solving Equation (1) using the CEF parameters for the cerium ion: $\Delta_{1}=0.043$ eV, $\Delta_{2}=0.066$ eV, $N_{0}=N_{1}=N_{2}=2$, and bandwidth D=4 eV. The temperature-dependent renormalized coupling constant $g_{R}\left(T,g\right)$ is also derived from Equation (1) using the same parameters. For a given *g*, the corresponding $T_{K}\left(g\right)$ is computed and substituted back into Equation (1). For a given temperature *T*, D=T is set and the equation is solved numerically, now treating *g* as a variable, to find $g=g_{R}\left(T,T_{K}\left(g\right)\right)=g_{R}\left(T,g\right)$.

Substituting the computed values into Equation (5), and using $E_{K}\left(g\right)=\frac{T_{K}\left(g\right)}{11600}$ eV/K, we solve for $T_{N}\left(g\right)$ to obtain the T–g phase diagram for CeRu_2_Ge_2_. The best agreement with the experimental T–p phase diagram is achieved after applying a linear transformation: g(p)=a+b·p, with the coefficients $a=23\;\times \;10^{-3}$ and $b=1.583\;\times \;10^{-3}$ GPa^−1^.

A similar procedure is applied to EuCu_2_(Ge_1−*x*_Si_*x*_)_2_, where the T–x phase diagram is computed by treating the Si concentration *x* as the main variable instead of pressure. For S=7/2 $\left(N_{0}=8\right)$ and using D=4 eV, z=6, and $F\left(\eta \right)=-8.514\;\times \;10^{-4}$, the RKKY energy becomes: $E_{RKKY}\left(x\right)=18.20\cdot x^{2}$ eV. The best fit to experimental data is obtained using a coupling constant scaled as: $g\left(x\right)=\left(10+17x\right)\;\times \;10^{-3}$.

Finally, we provide a rough estimate of the phase boundary between the AFM and the FL regions of the phase space, where $T_{N}\ll T_{K}$, i.e., close to the quantum critical point. We approximate the free energy of the FL phase as

(6) $$\begin{array}{c} F_{0}=E_{c}+E_{f}-E_{K}-TS_{0}, \end{array}$$

where $S_{0}≃T\gamma ≃\left(\pi^{2}k_{B}/3V_{0}\right)\left(T/T_{K}\right)$ is the entropy of heavy fermions in the FL regime and $V_{0}$ is the unit cell volume [46]. The condition $F_{0}=F_{N}$ gives $E_{RKKY}=E_{K}+T_{N}S_{0}\left(T_{N}\right)$, such that

(7) $$\begin{array}{c} T_{N}^{2}≃T_{K}\left(T_{RKKY}-T_{K}\right). \end{array}$$

Assuming a critical pressure of $T_{N}\left(p_{c}\right)=0$ gives the approximate result $T_{N}\left(p\right)\propto \sqrt{p-p_{c}}$.

## 3. Discussion

Before presenting the results which show how the competition between Kondo and RKKY interactions gives rise to Doniach diagram, we discuss briefly the characteristic features of the Anderson model in various parts of the phase space, using the parameters relevant for CeRu_2_Ge_2_ and similar RE intermetallics [34,35].

At ambient or low pressure and above 400 K, the low-laying CF states of cerium ions are occupied with equal probability, so that the conduction electrons scatter on the six-fold degenerate LMs. This gives rise to the resistivity and thermopower which are logarithmic functions of temperature (with a negative slope) [34,35]. Below 400 K, the excited CF states depopulate, and around $T_{\Delta }≃350$ K there is a crossover to a new LM regime, where the 4*f* state behaves as an effective CF doublet. This LM–LM crossover is indicated in the resistivity and thermopower data by the high-temperature maxima (see Figures 2 and 3 in Ref. [34]). The thermopower maximum is particularly pronounced, as α(T) drops around $T_{\Delta }$ from positive to negative values. For $T_{K}<T<T_{\Delta }$, the Kondo scattering on effective CF doublets gives rise to the resistivity and thermopower which increase towards their low-temperature maxima. The associated Kondo scale, inferred from the low-temperature maximum of α(T) or ρ(T), is very small [35], such that $T_{K}\ll T_{RKKY}$. Thus, it is not surprising that at ambient or low pressure, the paramagnetic entropy of CeRu_2_Ge_2_ is removed at low temperatures by an AFM transition, as indicated by a large specific heat anomaly and the discontinuity in the slope of ρ(T) and α(T) at $T_{N}$. The magnetic moment of Ce ions in the ordered state is much smaller than of a free Ce ion, but our analysis shows that this reduction is a CF effect and it is not due to the Kondo screening.

The model calculations and the experimental data show that $T_{N}\left(p\right)$ increases gradually with pressure up to a maximum and then drops rapidly. For $T_{N}≃T_{K}$, a precise estimate of $T_{N}$ from transport data becomes difficult, because the two maxima which characterise α(T) and ρ(T) are merged at higher pressure into a single broad maximum, such that a weak discontinuity of the slope is difficult to measure. Above the critical pressure, $p_{c}≃$ 6 GPa, we have $T_{N}<T_{K}$ and Kondo effect inhibits the formation of a magnetically ordered state.

At large pressure, $p\geq p_{c}$, we find $T_{RKKY}\ll T_{K}$, so that the RKKY interaction can be neglected throughout the LM regime. As temperature is reduced, the Kondo scattering leads gradually to the screening of the LM and, for $T\ll T_{K}$, the coherent state forms out of Kondo singlets [47]. The CeRu_2_Ge_2_ and similar compounds behave at low temperatures and $p\geq p_{c}$ as a heavy FL with an enhanced Pauli-like susceptibility χ, and a large specific heat coefficient, $\gamma =C_{V}/T$. The calculations for the periodic Anderson model show [46,47] that the enhancement of χ and γ scales with $T_{K}$, i.e., Kondo temperature provides the relevant energy scale at high and low temperatures. In the FL region of the phase space, the transport coefficients are given by simple powers of $T/T_{K}$ and the system is characterised by various universal ratios, like the Wilson ratio χ/γ, the Kadowaki–Woods ratio ρ/γ or the *q*-ratio α/γ.

The comparison between the experimental and theoretical Doniach diagrams of CeRu_2_Ge_2_ is provided by Figure 1, where the dashed line shows $T_{K}\left(p\right)$ calculated by the scaling theory and the full line is $T_{N}$. The values of g(p) used in the calculations are given on the upper abscissa and $T_{RKKY}$ is evaluated for the lowest CF doublet (S = 1/2), with D = 4 eV, z = 6, and $F\left(\eta \right)=-9.824\;\times \;10^{-4}$.

The gradual increase in $T_{N}\left(p\right)$ above the ambient pressure is due to the fact that $T_{K}\left(p\right)$ is exponentially smaller than $T_{RKKY}\left(p\right)$, so it can be neglected in Equation (5) (see the inset in Figure 1). Above a certain pressure, the exponential growth of $T_{K}\left(p\right)$ brings $T_{N}\left(p\right)$ to a maximum before reducing it sharply to zero. The asymmetric shape of $T_{N}\left(p\right)$ is due to different functional forms of $T_{K}\left(p\right)$ and $T_{RKKY}\left(p\right)$.

The scaling solution of an eight-fold degenerate Anderson model explains the phase diagram of EuCu_2_(Ge_1−*x*_Si_*x*_)_2_, where Silicon doping gives rise to the chemical pressure which drives this system from an antiferromagnet to a valence fluctuator [17,33]. The comparison with the experiment is shown in Figure 2, where $T_{N}$ (full line) and $T_{K}$ (dashed line) are plotted versus Si concentration (lower abscissa). The corresponding values of g(x) are given on the upper abscissa and the calculations are carried out following the same steps as in the case of CeRu_2_Ge_2_, taking S = 7/2, D = 4 eV, z = 6, and $F\left(\eta \right)=-8.514\;\times \;10^{-4}$. The theoretically calculated phase boundary in Figure 2 exhibits the same generic features as the experimental one. Note, our calculations take into account the spin–flip scattering of *c*-electrons on the eight-fold degenerate Eu^2+^ (4$f^{7}$) ions but neglect the fluctuations between Eu^2+^ and Eu^3+^ (4$f^{6}$) configurations. These fluctuations become important for large Si concentration, but to include them one would have to go beyond the scaling theory and consider a modified Hamiltonian which includes the Falicov–Kimball term [48].

We should also mention some important limitations that one should be aware of when analysing the experimental data in such a simplified way. The phase diagram is obtained by considering the competition between the Kondo and RKKY interactions, but more complex many-body effects, such as valence fluctuations or multipolar interactions, are neglected, even though they may be important near quantum critical points. Furthermore, the scaling approach used to derive the renormalized coupling constant assumes a paramagnetic background which is no longer satisfied once a long-range order is established. The assumed linear dependence of the exchange coupling constant on external control parameters (pressure or doping) fits well with the experimental data. However, the underlying electronic structure may be more complex and involve nonlinear changes in hybridisation, bandwidth, or crystal field under pressure or substitution. The method is based on phenomenological parameters and does not consider the data obtained from the electronic structure calculations. A more realistic treatment [22,24] takes an input from density functional theory or dynamic mean-field theory to evaluate the change in the density of states and hybridisation strength under pressure or doping. Disorder effects, which are particularly relevant in chemically substituted systems such as EuCu_2_(Ge_1−*x*_Si_*x*_)_2_, are also not taken into account. Due to the local variations in the chemical environment, the disorder can affect both the Kondo and RKKY energy and thus change the phase diagram. The comparison with the experiment is further complicated by the fact that the characteristic temperatures obtained from various experiments on EuCu_2_(Ge_1−*x*_Si_*x*_)_2_ differ by a factor of 2 or 3 and the values of $T_{K}$ inferred from the experimental data have a large error bar.

## 4. Conclusions

We described the phase diagram of heavy fermions with RE ions using the scaling solution of the periodic Anderson model. At high temperatures, we find that the system is in the LM phase with large paramagnetic entropy and that it is completely characterised by its Kondo temperature. Using the scaling law, we estimated the dependence of the coupling constant on the control parameter (pressure, doping, or magnetic field) and found $T_{RKKY}\left(p\right)$ and $T_{N}\left(p\right)$. The competition between the on-site Kondo coupling and the off-site RKKY coupling determines the mechanism by which the compound removes the paramagnetic entropy at low temperatures, i.e., it determines whether the ground state is a heavy Fermi liquid or an antiferromagnet. The huge effect of the control parameter on the ground state is explained by the differences in $T_{K}\left(p\right)$ and $T_{RKKY}\left(p\right)$ considered as functions of the control parameter.

In summary, despite its simplicity, our theoretical approach captures the main experimental features shown by the phase diagram of CeRu_2_Ge_2_, EuCu_2_(Ge_1−*x*_Si_*x*_)_2_ and other heavy fermions in which the coupling constant is an increasing function of the control parameter.

## Data Availability

The original contributions presented in this study are included in the article material. Further inquiries can be directed to the corresponding authors.
